# Supplementary material for: LncRNA SChLAP1 Promotes Cancer Cell Proliferation and Invasion Via Its Distinct Structural Domains and Conserved Regions
Source: J Mol Biol. Author manuscript; Available in PMC 2026 Jan 29. (PMC12854252; doi:10.1016/j.jmb.2025.169350)
Supplement: supplementary material Oh_JMB [file NIHMS2138646-supplement-supplementary_material_Oh_JMB.docx]

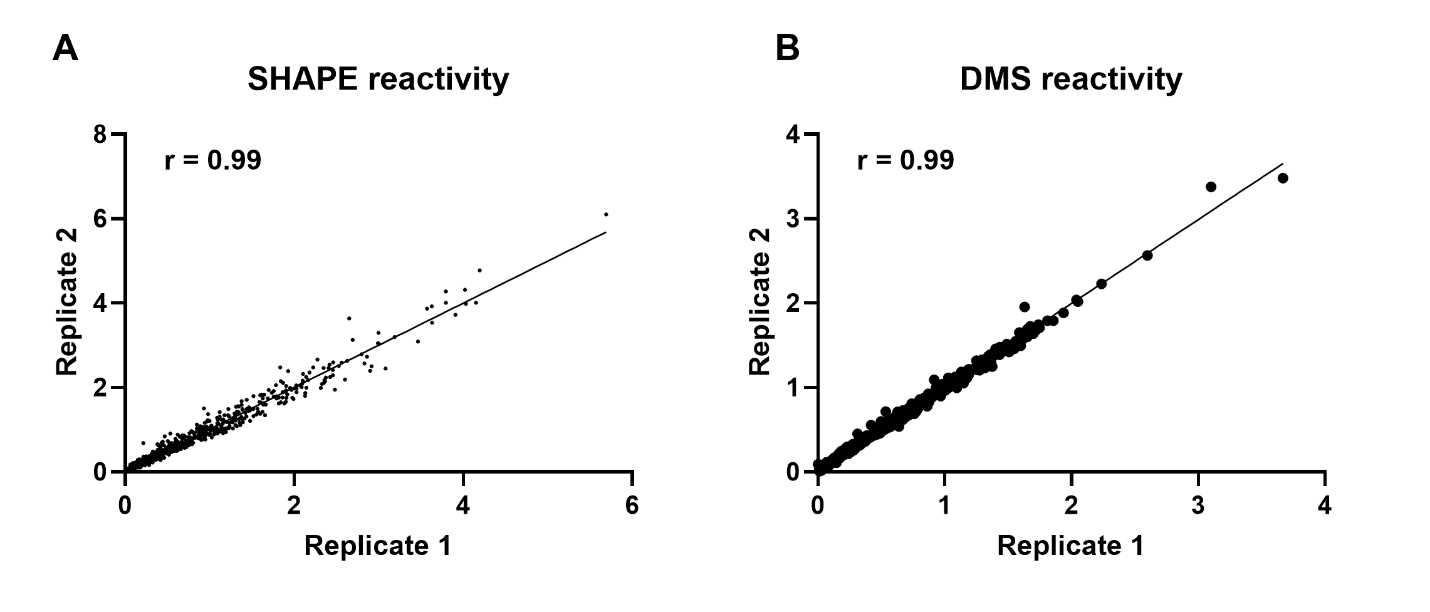


**Supplementary Figure S1.** Chemical probing of SChLAP1 in 25 mM Mg^2+^ folding buffer. SHAPE reactivity (A) and DMS reactivity (B) results were reproducible among different biological replicates. SHAPE reactivities (A) and DMS reactivities (B) at each nucleotide position were plotted and compared between the two biological replicates. Pearson correlation coefficient (*r*) values are indicated.


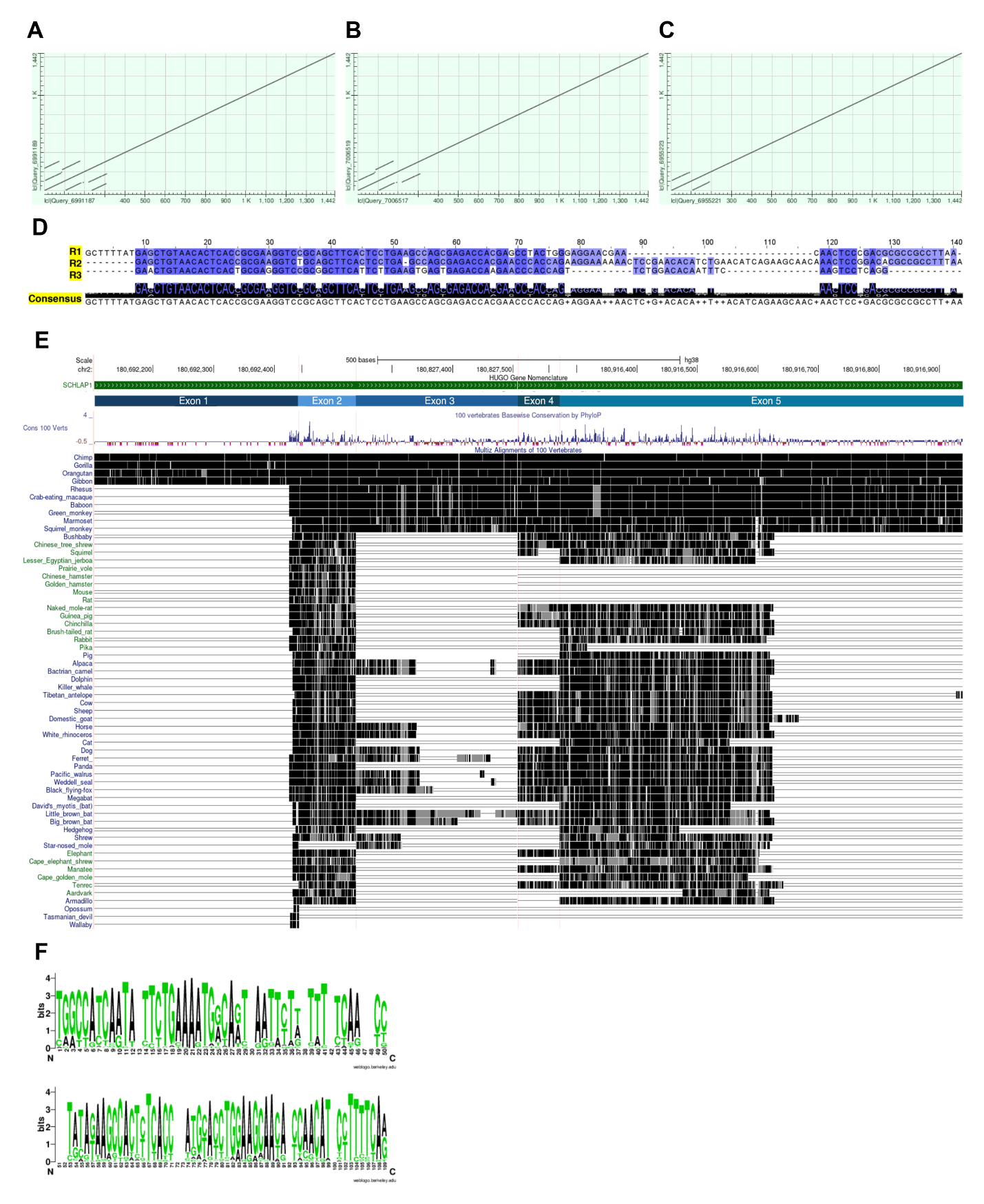


**Supplementary Figure S2.** Dot plots of BLAST self-alignment of SChLAP1 sequences with a setting of “somewhat similar (A),” “more dissimilar (B),” and “highly similar (C)”. Comparison of the SChLAP1 sequence with itself revealed the presence of several repetitive sequences with varying lengths and similarities. (D) Multiple sequence alignment by Clustal Omega revealed that the first exon of SChLAP1 consists of three repeat regions, including Repeat 1 (R1; nucleotides 1–110), Repeat 2 (R2; nucleotides 111–242), and Repeat 3 (R3; nucleotides 243–338) [1]. (E) Multiz alignment was performed for each exon, using basewise conservation scores across 100 vertebrate species as calculated by PhyloP. Black-colored regions indicate conserved sequences. (F) Graphical representation of conserved nucleic acid sequences of SChLAP1 (Exon 2: UGCCAUCAAUAUUCUGAAAAUGGCAGUGAUUUUUAUUCAACCUGUAUAAGGCACUUUCACCAUGUACCUGGAAGCAACAUCUACAUCUUUUUCAG). Multiple highly conserved motifs were identified throughout the second exon, including ‘TGGCCATCAATA’ (nucleotides 1-12), ‘TTCTGAAAATGGCAGT’ (nucleotides 14-29), ‘TATAGAAGCCACTCTCACC’ (nucleotides 53-71), and ‘ATGCACCTGGAAGCAACA’ (nucleotides 74-91). The height of each stack represents the degree of sequence conservation.


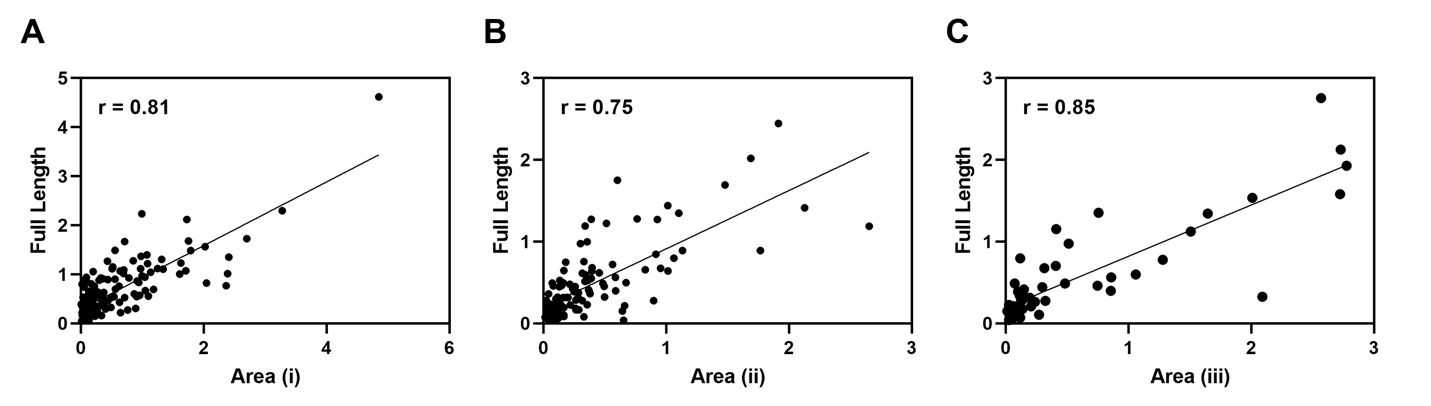


**Supplementary Figure S3.** (A-C) Scatter plots comparing SHAPE reactivities of Area (i) through (iii) (see Figure 5) with the corresponding region in the full-length SChLAP1. The data represent the average of two biological replicates. SHAPE reactivities at each nucleotide position were plotted and compared between the two biological replicates. Pearson correlation coefficient (*r*) values are indicated.


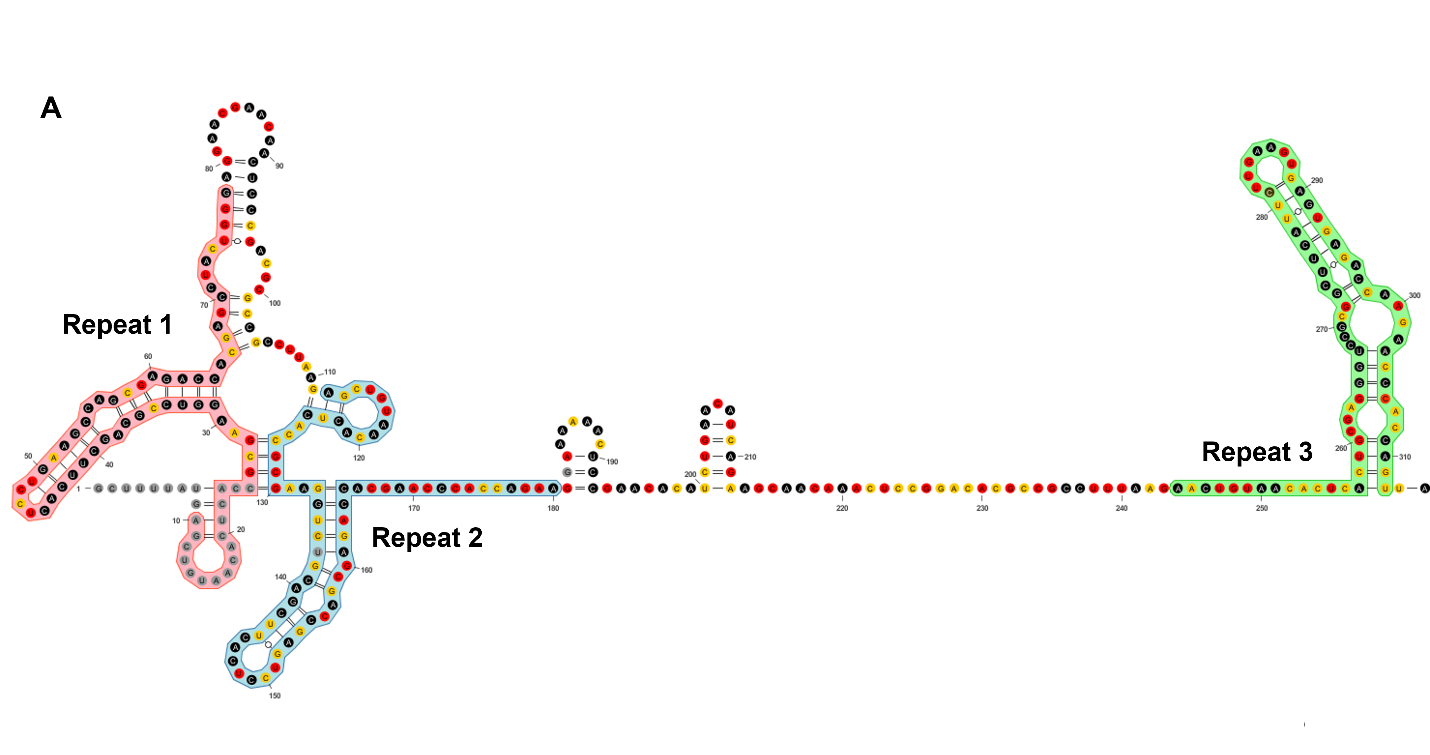


**Supplementary Figure S4.** Sequential and structural analysis of the repeats. Each of the three repeats is highlighted using different color schemes.


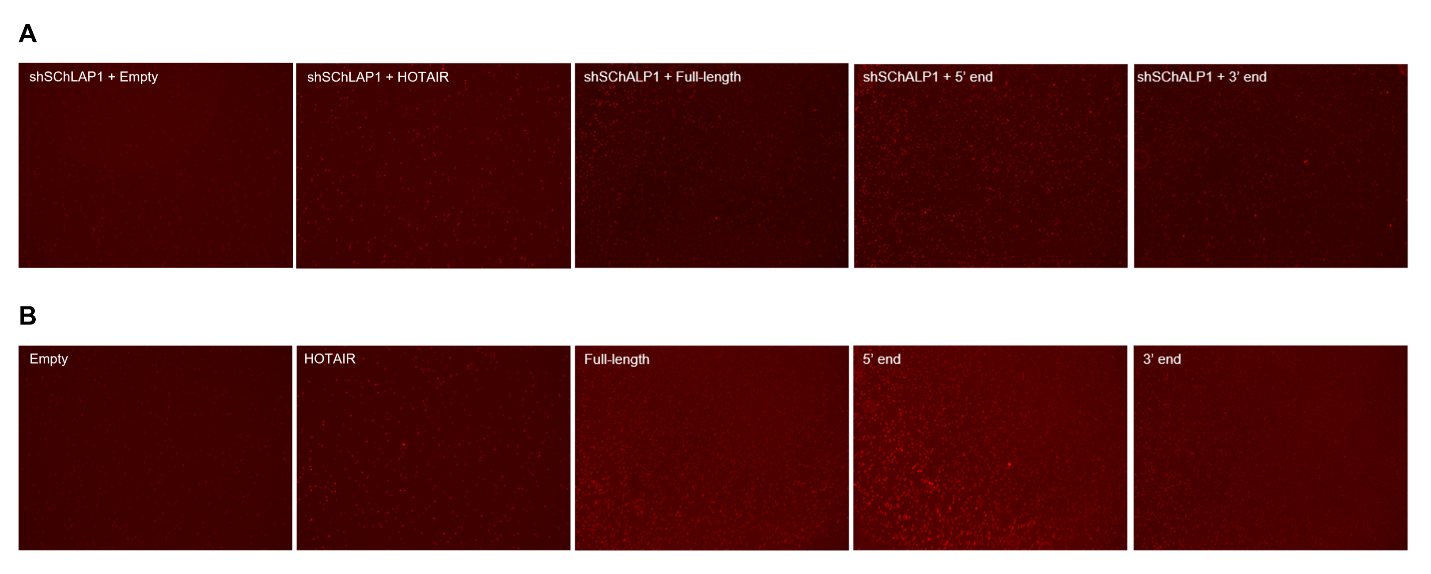


**Supplementary Figure S5.** Representative images of transwell invasion assays in LNCaP (A) and 22Rv1 cells (B). Cell invasion was assessed by averaging data from three randomly selected fields.


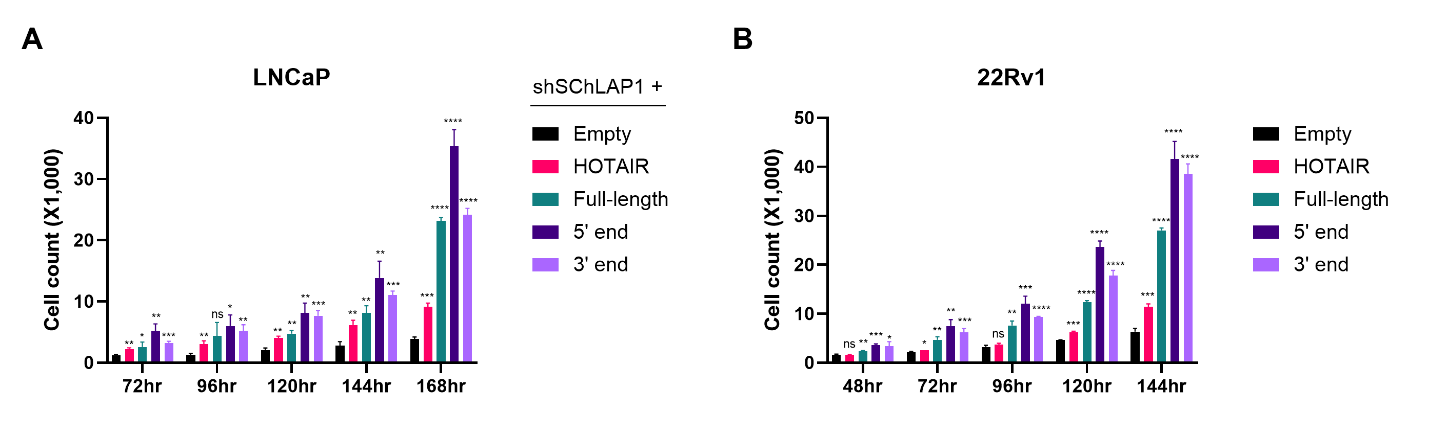


**Supplementary Figure S6.** Cell proliferation was measured by counting cells upon overexpressing the 5’ or 3’ end of SChLAP1 in LNCaP (A) and 22Rv1 cells (B). These plots are bar graphs depicting the data shown in Figures 6H and 6I.

**Supplementary Table S1.** Primer sequences used in this study.

| Site-directed mutagenesis | |  |
| --- | --- | --- |
| Target | **Forward Primer** | **Reverse Primer** |
| Fragment 1  (Nucleotides 222-651) | GACACGCCGCCTTTAAGAAC | CTTTCTCCATCCCGCTCCTC |
| Fragment 2  (Nucleotides 956-1428) | TCCATCAAGTCCTGGCTAACC | AATGGGCTCACAGTTCCACG |
| pLenti-GIII-3'-end | CTGAATTTCCATCAAGTCCTGGC | ATTTATAAGTGAAAGAGGTTTAATGG |
| *in vitro* chemical probing combined with mutational profiling | | |
| Target | **Forward Primer** | **Reverse Primer** |
| Full-length SChLAP1 | GCTTTTATGAGCTGTAAC | ATTTATAAGTGAAAGAGGTTTAATGG |
| Fragment 1  (Nucleotides 222-651) | GACACGCCGCCTTTAAGAAC | CTTTCTCCATCCCGCTCCTC |
| Fragment 2  (Nucleotides 956-1428) | TCCATCAAGTCCTGGCTAACC | AATGGGCTCACAGTTCCACG |
| *in vivo* chemical probing combined with mutational profiling | | |
| Target | **Forward Primer** | **Reverse Primer** |
| Nucleotides 1-740 | GCTTTTATGAGCTGTAAC | GCCTCTTGGGTTCACCATCT |
| Nucleotides 586-1119 | CCTCCCTGAAGAAGCTGAATATC | CACAGCCAAACCATATCACATG |
| Nucleotides 958-1436 | CTGAATTTCCATCAAGTCCTGGC | ATTTATAAGTGAAAGAGGTTTAATGG |
| RT-qPCR |  |  |
| Target | **Forward Primer** | **Reverse Primer** |
| Full-length SChLAP1 | TGGACACAATTTCAAGTCCTC | CATGGTGAAAGTGCCTTATAC |
| 5'-end SChLAP1 | TGGACACAATTTCAAGTCCTC | CATGGTGAAAGTGCCTTATAC |
| 3'-end SChLAP1 | ACCAATGTTCACTGTGAAGGA | GGGGACACAGCCAAACCATA |
| GAPDH | GAAGGTCGGAGTCAACGG | ACATGTAAACCATGTAGTTGAGGT |
| MMP9 | CGTCGGTCCGTCCGCTA | GGGAACATCCGGTCCACCT |
| MMP14 | AGTGGATAGCGAGTACCCCA | TTCTCTCAGCGACACCTTCC |
| VEGF | CCTGTTCCGAGGTTGCCCT | AGGACCAACAGCCACTATGAG |

**References**

1. Sievers, F., et al., *Fast, scalable generation of high‐quality protein multiple sequence alignments using Clustal Omega.* Molecular systems biology, 2011. **7**(1): p. 539.
